# Psychiatric Admission Documentation at Leverndale Hospital, Glasgow

**DOI:** 10.1192/bjo.2024.622

**Published:** 2024-08-01

**Authors:** Cecilia Rafters, Wai Lan Imrie

**Affiliations:** Leverndale Hospital, Glasgow, United Kingdom

## Abstract

**Aims:**

To ensure psychiatry admission assessments are well-documented and available via EmisWeb clinical notes system.

To assess whether replacing the clerk-in booklet with a digitalised version affects documentation of psychiatry admission assessments.

The standard was use of the current admission template with information under each heading. In addition, a Clinical Risk Assessment Framework for Teams (CRAFT) tool should be completed by the admitting doctor. Legal status, observation level and time-out status should be recorded.

**Methods:**

A retrospective full-cycle audit of the first twenty patients admitted to Leverndale Hospital, Glasgow starting 1st Dec 2022, then 1st April 2023 for the second cycle. Patients were identified using EMISWeb admission dates and information from Medical Records. Data was taken from the EMISWeb record and analysed using Microsoft Excel – if incomplete paper notes were checked.

Use of the admission template and presence of meaningful content (excluding “n/a” and similar) under each heading was recorded. Data was collected on the documentation of legal status, observation level, time-out status, and CRAFT risk assessment by the admitting doctor. Perinatal psychiatry admissions and transfers between psychiatric units were excluded.

**Results:**

Medicines reconciliation was absent in 55% and allergies in 70%. CRAFT risk assessment was completed by the admitting doctor in 55% of cases.

Following round 1, a digitalised admission proforma was introduced, replacing the traditional admission booklet.

There was little improvement in usage of the admission proforma with the introduction of the digitalised version (80% vs 75% previously). Notably, the rates of CRAFT risk assessments by the admitting doctor fell in this 2^nd^ cycle – from 55% to 40%.

Recording of allergy status (55% from 30%) and medications (95% from 45%) seemed to improve. Importantly quality of content was not appraised – many medication entries consisted of “as per HEPMA (electronic prescribing system)”.

100% of admission documentation was now available on EMISWeb, preventing information loss and allowing remote access from Community Mental Health Teams.

**Conclusion:**

The introduction of a digitalised admission template had little impact on use of the proforma. However it meant that 100% of admission information was available digitally, versus 85% previously.

In both cycles the CRAFT risk assessment was often not completed by the admitting doctor. Given risk assessment is a key reason for “clerking in” psychiatric inpatients, this could be a focus for future quality improvement work.

In the second cycle, recording of medications and allergy status had improved significantly. Given small sample sizes, this may be due to chance.

